# “Health is Just the Basic Requirement for Optimal Performance and Winning”: Stakeholders’ Perceptions on Testing and Training in Competitive Alpine Skiing, Snowboarding and Freestyle Skiing

**DOI:** 10.1007/s40279-024-02106-0

**Published:** 2024-09-13

**Authors:** Oriol Bonell Monsonís, Peter Balsiger, Evert Verhagen, Vincent Gouttebarge, Jörg Spörri, Caroline Bolling

**Affiliations:** 1 Department of Public and Occupational Health, Amsterdam Movement Sciences, Amsterdam Collaboration on Health and Safety in Sports, Amsterdam UMC, University Medical Centres-Vrije Universiteit Amsterdam, Location VUmc, De Boelelaan 1117, 1081 HV Amsterdam, The Netherlands; 2 Amsterdam Movement Sciences, Musculoskeletal Health, Sports, Amsterdam, The Netherlands; 3 Sports Medical Research Group, Department of Orthopaedics, Balgrist University Hospital, University of Zurich, Zurich, Switzerland; 4 University Centre for Prevention and Sports Medicine, Department of Orthopaedics, Balgrist University Hospital, University of Zurich, Zurich, Switzerland; 5 Amsterdam UMC location University of Amsterdam, Department of Orthopedic Surgery and Sports Medicine, Amsterdam, The Netherlands; 6 Section Sports Medicine, Faculty of Health Sciences, University of Pretoria, Pretoria, South Africa

## Abstract

**Background and Objective:**

Competitive alpine skiing, snowboarding and freestyle skiing, all different in nature and risks, are known for their high injury and illness burden. Testing measures and training methods may be considered for athletes’ preparation to support performance enhancement while safeguarding their health. We explored the perspectives and perceptions of competitive alpine skiing, snowboarding and freestyle skiing stakeholders regarding testing and training practices in their competitive snow sports.

**Methods:**

We conducted an exploratory qualitative study based on grounded theory principles through 13 semi-structured interviews about testing and training practices with athletes, on-snow and off-snow coaches, managers and healthcare providers from different national teams. The interviews were inductively analysed through a constant comparative data analysis.

**Results:**

Participants described winning as the end goal of testing and training practices, which requires athletes to perform in their best condition. To do so, they mentioned two main targets: performance enhancement and health protection. Participants acknowledged health as a premise to perform optimally, considering testing and monitoring approaches, goal setting, and training to support and protect athlete performance. This continuous cyclic process is driven by communication and shared decision making among all stakeholders, using testing and monitoring outputs to inform goal setting, training (e.g. on-snow and off-snow) and injury prevention. Such an approach helps athletes achieve their goal of winning while being fit and healthy throughout their short-term and long-term athletic career development.

**Conclusions:**

The ultimate goal of testing measures and training methods in such competitive snow sports is winning. Performance enhancement and health protection act as pillars in systematic, tailored and flexible processes to guarantee athletes’ best preparation to perform. Moreover, athletes’ assessments, goal setting, monitoring tools, open communication and shared decision making strongly guide this cyclic process.

**Supplementary Information:**

The online version contains supplementary material available at 10.1007/s40279-024-02106-0.

## Key Points

**Table:** 

Qualitative research methods can be employed to understand testing and training practices and gain insight into the challenges and opportunities within competitive snow sports environments.
Performance enhancement and health protection goals are the backbone of the ultimate design and development of training plans. These goals involve a cyclic process guided, modulated and influenced by the athlete’s assessment and monitoring.
Goal setting and training planning are collaborative and athlete-centred processes that require open communication, effective teamwork and shared decision making among coaches and medical and technical staff around the athlete, eventually leading to the training plan.

## Background

Competitive alpine skiing, snowboarding, and freestyle skiing, despite their different features and nature among competitive snow sports, demand high levels of physical fitness, technical mastery and mental abilities [1]. Athletes participating in such competitive sports are exposed to high loads (e.g. training and competition loads, race calendar congestion, psychological load and travel). In turn, by their training programme, athletes and their support staff seek for methods to increase fitness and, consequently, improve performance over time [1, 2]. However, a poor load management, in combination with the increasingly saturated race calendars, may affect an athlete’s health [3]. Specifically, testing measures and training methods may be considered for athletes’ preparation to support performance enhancement while protecting their health [1, 4–6]. Thus far, it has been advocated that athletes need training to reach their best performance level while testing data is important because it can inform future training structures and plans [4, 6, 7]. Similarly, the International Ski and Snowboard Federation (FIS) recently adopted the Athletes’ Health Unit, intending to provide a framework to protect physical and mental health while allowing for long-term high performance [8]. In this regard, testing and training approaches are embedded within the athlete-related pillars of this framework.

On the one hand, sport-specific tests guide the training process by predicting an athlete’s current performance level and evaluating performance progress [9, 10]. For example, Austrian ski and snowboarder athletes are tested across different areas, such as aerobic and anaerobic capacity, muscular strength and power, and neuromuscular function (e.g. balance, agility and coordination) [9]. Furthermore, it has been acknowledged that regular testing can contribute to prevent injuries and overtraining by tracking training adaptions [6, 9]. On the other hand, training methods aim to increase and optimise athletes’ performance to cope with the specific demands of their sport [4, 11]. Distributed across two main periods (e.g. preparation and competition periods), most training programmes include strength, power and neuromuscular training, technical training, and often involve cross-training in other sports [1, 7, 12]. In this connection, an integral approach to training load provides insights into training stress and response through training metrics, including monitoring variables related to external loads (e.g. training and race time, frequency and type, runs, strength and neuromuscular function) and internal loads (e.g. perception of effort and sleep) [3, 12]. Assessing load measures allows for the evaluation of an athlete’s adaptation, fatigue and recovery status, adjustment of an individual training programme, its impact on performance, and minimising the risk of injury and illness [12, 13]. Hence, an optimal training load management would yield performance and health benefits, acknowledging that both the temporal context and environment influence an athlete’s load and load capacity [4]. In respect to injury prevention, the training load has been suggested to drive the athlete towards or away from an injury [4, 13, 14]. Taken together, given the evolution of equipment, changing environmental factors (e.g. weather and snow conditions), and the complexity and nature of these snow sports disciplines, multiple factors come into play in relation to athletes’ performance and health [1, 9, 15].

Within the specific context of these competitive snow sports, testing and training may be a particular challenge. Moreover, these sports take place under extreme and difficult to standardise outdoor conditions. Therefore, testing and training become even more relevant because of these inherent features and potential limitations of the sports setting, which also includes on-snow and off-snow season periods, challenging and diverse snow and weather conditions, and busy schedules [1]. However, little is known within this context about the influence and impact of on-snow and off-snow season testing measures and training methods on optimising performance while ensuring athlete safety within the representative real-life settings. Additionally, there is a lack of understanding of how contextual and environmental factors influence both testing and training [4, 16]. Further, on-snow and off-snow periods vastly differ, as testing and training practices within these periods may do too. In relation to an athlete’s health status, an injury/health and performance relationship through a risk grading system between both constructs has been advocated [17]. Thus, assessing athletes through testing and monitoring may contribute to the identification of potential injury risks and health factors to develop further tailored performance-oriented training plans for physical and mental fitness [1, 6, 7, 9, 18]. Likewise, there is a need for snow sport-specific training approaches against *one-size-fits-all* practices to train and prepare athletes conveniently [1, 5, 7, 13]. Altogether, both testing and training processes can assist, through health protection (e.g. injury and illness prevention), the overall well-being and longevity of athletes’ involvement in alpine skiing, snowboarding and freestyle skiing.

The current literature acknowledges and highlights the relationship of testing and training, through load monitoring, with injury risk prevention [6, 11, 19]. However, specific snow sports-specific research in these areas is limited and all the knowledge on these areas comes from personal experience and anecdotal information or remains protected and unpublished. Despite the limited research within the field, the great potential of testing and training interventions from a prevention perspective has been highlighted. To date, mainly in young athletes, testing measures have targeted physical-related and biomechanical-related aspect (e.g. neuromuscular coordination, strength, muscular activation patterns, and dynamic postural stability tasks) [20–23]. Regarding injury prevention training programmes, the focus has been on home exercises addressing alpine skiing-specific injury mechanisms [24] and indoor and outdoor exercises combined with an educational intervention for preventing anterior cruciate ligament injuries [25]. Yet, there is a need to understand and comprehend testing and training practices within the competitive snow sport context and how contextual and environmental factors (e.g. experience, communication, shared responsibilities, busy calendars, race venues) primarily influence athletes, coaches, and team staff when designing and performing their current testing and training practices.

Accordingly, the voices of athletes and all stakeholders may help to gain insight into how and why testing measures and training methods are used within this context from a performance and safety perspective [16, 26, 27]. Thereby, qualitative methods are one way to increase the meaning and knowledge of such approaches, yielding a more in-depth outlook on the topic as they are the main end users of interventions on a daily basis [28]. Therefore, this qualitative study explored the perspectives and perceptions of stakeholders from competitive alpine skiing, snowboarding and freestyle skiing regarding testing and training practices in their setting.

## Methods

### Study Design

This study used an exploratory qualitative research design applying grounded theory (GT) principles [29, 30]. Grounded theory is a systematic and inductive method that focuses on building new theories from data to generate concepts and categories from the data. Our qualitative research focused on processes and interactions by interpreting and generating connections from various perspectives and participants’ voices within an interpretive paradigm [31]. The results are reported according to the Consolidated Criteria for Reporting Qualitative Research guidelines (Electronic Supplementary Material [ESM]). [32]

### Recruitment and Participants

Potential competitive snow sport participants were invited to participate in the study through an invitation e-mail by one of the researchers (JS), who worked jointly with FIS. The eligibility criteria included to be > 18 years old, professionals working within their competitive snow sport setting, and to be able to communicate in English or German. Participants were provided with an information letter with a written explanation of the study, informed about their voluntary involvement, and reminded about anonymity and confidentiality. Following the acceptance to participate, participants were further contacted by the research team.

Considering that testing and training are approached and conducted by different professionals in competitive snow sports, and based on the maximum variation sampling method [33], we aimed to ensure that the diverse groups of participants represent different roles, disciplines and countries, allowing a broader outlook of the topics from multiple perspectives, backgrounds and experiences. The sample consisted of 13 (four female and nine male) competitive snow sports athletes (*n* = 2), head coaches and managers (*n* = 3), on-snow coaches (*n* = 1), off-snow strength and conditioning (S&C) coaches (*n* = 3), physiotherapists (*n* = 3) and sports psychologists (*n* = 1). Participants, with less and more experience within snow sports, worked at the elite level in alpine skiing (*n* = 9), snowboarding and free skiing (*n* = 4) on the World Cup and European Cup circuits of different FIS snow sports disciplines. Among all competitive snow sports, we sought to involve competitive alpine skiing, snowboarding and freestyle skiing stakeholders given that these disciplines showed the greatest injury incidence and severity at the Beijing 2022 Winter Olympics Games [34]. Moreover, included participants were from the national teams of Switzerland (*n* = 3), Austria (*n* = 2), Germany (*n* = 2), Canada (*n* = 1), Finland (*n* = 1), Japan (*n* = 1), New Zealand (*n* = 1), Norway (*n* = 1) and the USA (*n* = 1). To guarantee confidentiality, no additional demographic details are presented.

### Data Collection

The data were collected through online semi-structured interviews from May to August 2022. All interviews were conducted by video call by one researcher (PB) in English or German, depending on the participants’ preferences. Thus, seven of the 13 interviews were conducted in English, and six were conducted in German. The interviews were audio-recorded. The mean length of the interviews was 46 min (range 34–71 min). All data were pseudoanonymised; unique reference numbers were used for each participant. During interviews, handwritten notes were taken by the interviewer [35]. The interview guide covered questions about their perspectives regarding training goals, planning and execution of training, testing measures, future perspectives of training and testing, and the roles of injury prevention and mental aspects within testing and training practices. Before the start of the study, the interview questions were tested in a pilot interview and refined afterwards. This pilot interview was not included in the sample. Throughout the ongoing process of data collection, we also used theoretical sampling to include new participants with different roles and disciplines to obtain different perspectives and a more in-depth understanding of emerging concepts [33], as well as questions were added and adjusted in response to the newly gained information. The topic list is presented in the ESM. After 13 interviews, no conceptually meaningful data emerged from the interviews as the same constructs were repeated in variation and meaning, indicating saturation [36]. All participants provided verbal informed consent before their interviews. This study was performed in accordance with the standards of ethics outlined in the Declaration of Helsinki. The present study protocol was reviewed by the Cantonal Ethics Committee KEK Zurich (BASEC Nr. Req. 2021-01329) and was judged not to fall within the scope of the Human Research Act (HRA).

### Data Analysis

**Fig. 1:**
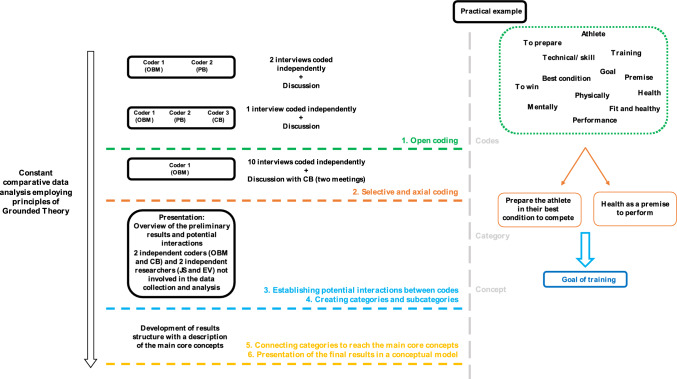
Step-by-step diagram of the data analysis process. On the *right-hand side*, there is an example of our coding process from codes to categories to eventually concepts

All interviews were transcribed verbatim and further inductively examined following a constant comparative data analysis [37], applying principles of GT, including open, selective and axial coding [29, 30]. To ensure consistency throughout the process, the interviews were analysed in their original language, and all the codes were processed in English. In parallel to data collection, collected data were analysed to inform the ongoing data collection [38]. During the coding process of every new interview, codes of previous interviews were compared, and if needed, codes were refined or merged. In the first stage, two researchers (OBM and PB) independently open-coded two interviews using ATLAS.ti software (Scientific Software Development GmbH, Berlin, Germany; version 8.4.5). The open coding is the initial stage of the comparative analysis with the aim of labelling the transcript data that described and conceptualised the dataset [30]. Then, they discussed their codes. OBM, PB and CB coded one additional interview independently to ensure coherence and consistency. Subsequently, codes and memos were discussed, and the analysis was refined in a team meeting (OBM, PB and CB). After reaching an agreement (e.g. removing themes when not supported by enough data, and merging related themes into a single main inclusive theme), one researcher (OBM) coded the remaining interviews (*n* = 10). Over two meetings between OBM and CB, in which the authors examined the emerging findings and potential interactions, an outline was introduced to two independent researchers (EV and JS) not involved in the data collection and the first steps of the analysis. During this meeting between the researchers (OBM, CB, EV and JS), codes, categories, and discrepancies were considered and compared until the categories of interest were settled for analysis. Consequently, preliminary results were developed from the analysis. Following the constant comparative data analysis, similarities, differences and connections were discussed to obtain the main concepts with a description of the core concepts (Fig. 1). In addition, a practical example of the data analysis process is provided in Fig. 1, where codes from the initial coding phase were merged into two categories and further integrated into one main concept.

### Reflexivity

The research team consisted of five male individuals and one female individual. OBM is a PhD candidate from Andorra and a sports physiotherapist with experience in alpine skiing injury prevention. PB is a Swiss human movement scientist and an athletic coach. EV is a Dutch sports scientist and epidemiologist with thorough expertise in injury prevention. VG is a French sports medical scientist, researcher and former professional athlete. JS is a Swiss human movement scientist with extensive experience in alpine skiing research and injury prevention. CB is a Brazilian sports physical therapist with experience in sports injuries and a postdoctoral researcher. Considering the researchers’ backgrounds, the analysis may have potentially been influenced by an overemphasis on aspects of injury prevention. Nevertheless, the diversity of perspectives and backgrounds represented by the authors supports the impartiality of our findings.

## Results

**Fig. 2:**
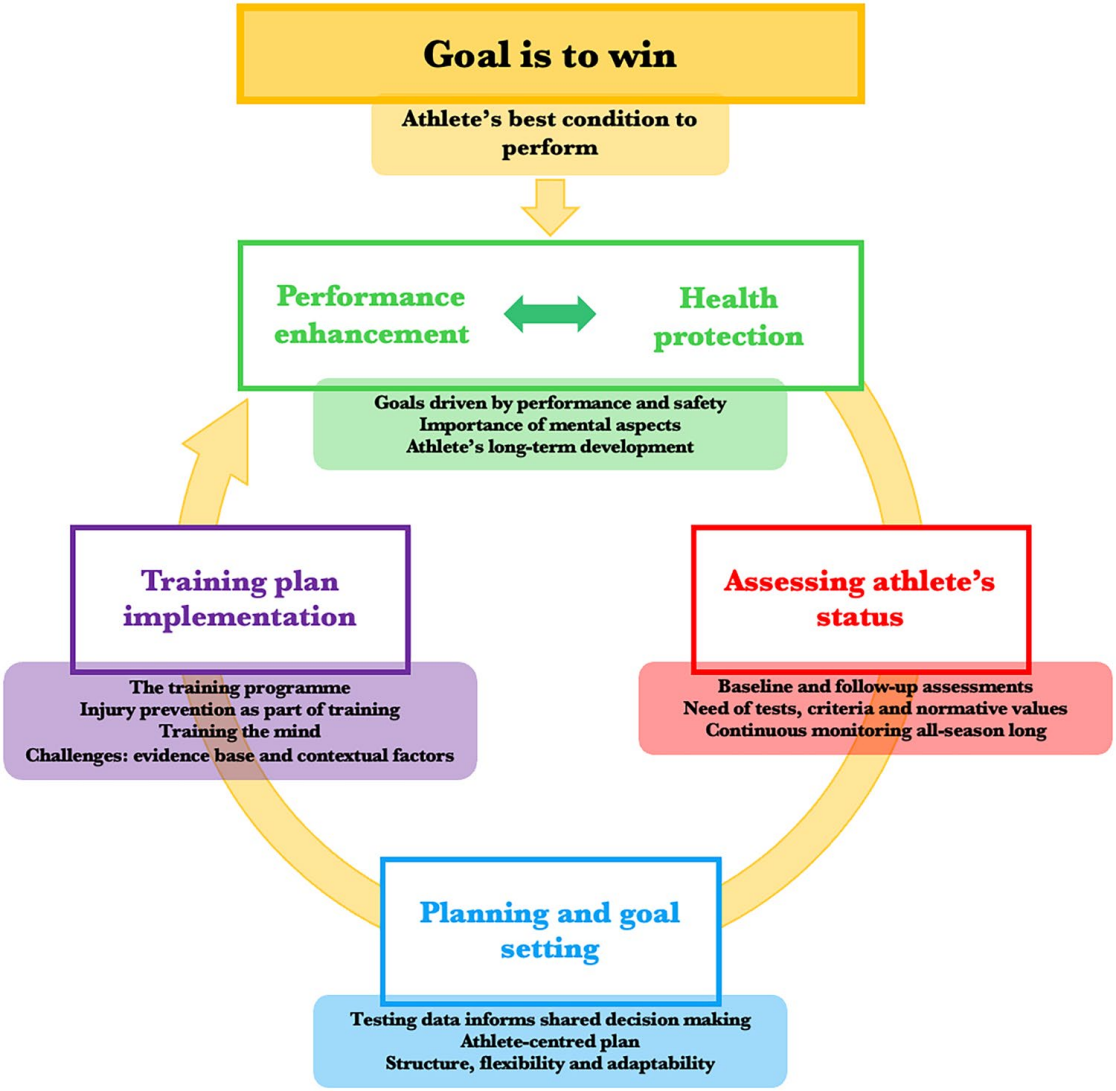
Sequence of winning in competitive snow sports: performance and health as pillars to achieve athletes’ best condition to perform. Based on our data analysis, this figure describes the systematic and cyclic approach of testing measures and training methods to ensure athletes’ preparation to perform optimally in competitive snow sports. The overarching goal of this process is winning (*yellow boxes*). Following a cyclic course, different factors are essential for supporting and helping athletes win: **a** providing strategies to enhance their performance while protecting their health (*green boxes*); **b** periodically assessing their physical and mental status (*red boxes*); **c** establishing a shared decision-making process driven by communication among all the team members that assists in goal planning and goal setting according to the athlete’s needs (*blue boxes*); and **d** eventually implementing a tailored physical and mental training plan, which is continuously nourished by the previous stages and feeds the successive athlete plans following the same pattern. These aspects are critical in the wheel of success and occur in the short-term (actual season) and long-term (athlete’s career) stages in competitive snow sports athletes. However, this development pathway encounters some limitations across all process steps

The main concept from the data analysis was that the ultimate goal of training is getting the athletes in their best condition to perform and win. Testing and training methods are conducted via a systematic and cyclic approach, with performance and health as pillars to achieve the athlete’s best performance. Figure 2 outlines the testing and training cycle supporting athletes throughout their short-term and long-term development. Our findings are presented according to the main concepts, related subcodes and quotes in Tables 1, 2, 3, 4, and these tables are structured based on our main findings and represented by multiple stakeholders.

**Table 1:** Concepts, subcodes and exemplary quotes on the ultimate goal of training is winning: balancing performance and health

Main concept	Subcode	Exemplary quote
Testing and training are needed to achieve the end goal of winning	Goals are driven by physical and mental health	“The main goal of training for athletes is performance, so, winning medals. […] There could be pressure from coaches, from parents, from themselves. They can feel a lack of support from the team, or they can feel very high support from the team, which is positive. There may be economic challenges and stress because they need to perform, so they may have to perform to make a living, which might be a pressure and an insecurity that they don’t have a stable income. So, I would say it’s probably possible to list 20 or 30 different possible mental stress factors. And they’re different, you know, among all athletes.” **Physiotherapist 1** “Above all, the goal is to get from A to B, from start to finish, as quickly as possible. To be the fastest at the end. […] I think in the long run, it’s very important that athletes stay healthy as early as possible because health is just the basic requirement for optimal performance. Only a healthy athlete brings his full performance.” **Physiotherapist 2** “Goals of training are to prepare athletes to perform the best they can, under the pressure of competition, without injuring themselves.” **Sports psychologist** “It’s trying to make them better free skiers and better snowboarders, getting them on snow in the best condition possible to learn new tricks. […] Generally focusing on the sport, trying to make them better at it, and trying to get them fit and healthy.” **S&C coach 1** “Health has actually been our top priority for several years, health and performance development. We simply believe that only healthy athletes can then really develop. […] The goals are, of course, individual, depending on the age group or the level of the athletes. We have different goals in the summer season, so for me, it was always important to develop the athlete physically and mentally. […] I think it’s important to have a plan, to structure it and follow it. Performance development and dealing with pressure are important, but always at the right time.” **Head coach/Manager 1** “Every athlete has to do it themselves. Stay healthy. Their health is a top priority so that way they can achieve their goals at all. […] The athletes know they are kind of putting their bodies at risk. They have to go full throttle. Otherwise, you are nowhere. You never win a race. But you have to do everything for it, and I would also like to recommend that they are aware of health. That is the top priority logically, and only then is success.” **Former athlete, and current S&C coach** “Because if there is pain somewhere, there is nothing left to do with skiing. The best (performance) values are useless. […] The athlete should be pain-free and get as good as possible through the season. Then, a lot has already been gained.” **S&C coach 2** “I think if you manage to go through a career without injury, then the chance that you'll be at the top is much greater than if you keep coming back.” **Former athlete, and current physiotherapist and S&C coach**
	The mental aspect is as important as the physical aspect	“In the World Cup, I would say that anyone can ski, and then everything has to come together. In the end, the pieces of the puzzle have to fit together. And the head is certainly a big deal.” **Former athlete, and current S&C coach** “The role of mental aspects in alpine skiing is huge. I mean, you cannot disconnect the mind from the body. One affects the other, and vice versa. And so, it does need to be considered. […] Because you can be as good as you want physically prepared, but if the mental side is not working well … I believe it is needed to perform at the highest level.” **Physiotherapist 3** “You would have to build up the body and the mindset alongside it. So, it’s still the two big pillars, but different levels, different problems, different goals between athletes. […] The more positive you are mentally, the faster and better you progress. And the better goals you set for yourself” **On-snow coach** “If it’s just the school stress for the young athletes, in which they are always under a stressful situation because they have school, they have exams, but they’re also supposed to train and ski. It’s definitely a mental challenge, and I think you shouldn’t underestimate that either if you’re no longer a student but are married or have unmarried children at home. I think sometimes it’s just a mental challenge. Being in Chile for 4 weeks and leaving your wife and children at home and being gone for so long is a psychological burden sometimes for the athletes.” **Physiotherapist 2** “There is a lot of pressure on athletes these days. It’s from the association, from the association's partners, and the media from time to time. Although we don’t exert any pressure, the pressure is exerted with the squad criteria. This is the first step where you put pressure on athletes.” **Head coach/Manager 1** “Being fully ready on day X and being able to deliver. That for sure is something. Fear is not allowed. So, if an athlete is scared, they don’t come forward. This is not for winning. […] You can practice that (being ready for day X) and take that with you into your daily training. So, how do you get there? What are your goals? Practice visualizations; you can build that up well. Then, well, a competitive sport is a competitive sport. You won’t get ahead if you don’t bring it with you or if you don’t learn it well enough.” **On-snow coach**
	Short-term and long-term goals of an athlete’s career development	“A large part of our training—it depends on what level—is to become an athlete. That means strengthening, but it also includes the mental side. It’s a big development process.” **On-snow coach** “Thinking long-term, but with the focus that the ultimate goal should be to get to the top of the world, this is World Cup.” **Head coach/Manager 1** “I’m focused on performance, I’m focused on the execution, in the pressure of competition, and I’m focused on getting them to a situation where they don’t push too hard, too fast at the wrong time.” **Sports psychologist** “Because in the end, it’s good to have a kid that can have pieces, but we need to have the whole business. One of our philosophies is to have a well-trained athlete who can do many tricks. He can do many things on the skis, but are they also a good dryland athlete? […] Can they move right and left, move up and down? Can they do different actions? Can they then take that into their skiing, and then we can make good skiers out of them? So, first good athletes, then good skiers.” **Head coach/Manager 2** “In youth athletes, the aim of training is more into development and being able to take steps in their sports with low risk of injuries. It is more a general conditioning and sort of building them up to manage the loads that will come in later years.” **Physiotherapist 1**

*S&C* strength and conditioning

**Table 2:** Concepts, subcodes and exemplary quotes on the essential role of assessing the athlete’s status

Main concept	Subcode	Exemplary quote
Why is assessing the athlete’s status fundamental?	What is the status quo of the athlete? Baseline and follow-up assessments	“It’s more to see where they’re at. Also, what things they’re good at and what things they are not so good at.” **Head coach/Manager 2** “They do the baseline physical testing twice a year, and the results from these tests will affect what is programmed later.” **Physiotherapist 1** “So, we have a testing protocol which we do several times throughout the year and during the off-season. […] The testing protocol slightly differs from what they’ll be doing during in-season. So, we try and get some data on where the athletes are at with certain physical qualities. And then try and kind of see the context behind those numbers a little bit for each athlete. They will use watches, […] and we’ll get a basic overview on the type of training that they have been doing.” **S&C coach 1** “We have certain testing measures; we have screening, and we do testing at baseline. Of course, the current state of the athlete is also recorded. Where their weaknesses are, where their strengths are, and which parameters could prevent him from their development in the ski area.” **Head coach/Manager 1** “We do the classic FMS, starting with all medical tests and then moving to specific and controlled things. […] Then we do strength tests, isometric strength tests, and muscular endurance tests for the core. Then, dynamic tests and jump tests. […] We have a baseline testing for any injuries, which is later our return-to-competition testing.” **Physiotherapist 2**
	There is a critical need for adequate tests, criteria and normative values	“There’s still a real lack of data. We still can’t say 100% to our athletes, “*This is important for you, so you need to do this”*, so we’re starting out with these tests, gathering these data and seeing how that might help.” **Head coach/Manager 3** “Until now, it has been very alpine skiing-centred. But you didn’t understand what you have to measure in freestyle, what values you need, how you measure it, and what is important and not.” **On-snow coach** “Testing is still really under-researched in our discipline, and we’re still trying to find the answers to what is important and essential to support the riders in doing what they do. And keep them injury-free and strong enough to do what they’re doing. So, this lack of information is the biggest trouble at the moment.” **Physiotherapist 2** “For the youth athletes, we rarely have baseline tests to compare with. So, if the athlete is injured, we are training to restore function and be able to come back to normal training. For the same aim of symmetrical function.” **Physiotherapist 1**
	Monitoring as the approach of repetitive testing all season long	“How was the sleep quality? How does he feel? What is his energy level? There is the training plan and we evaluate athletes before and after the snow training, how they felt, what they thought about the training and how stressful the training was. Then they do the same thing again in the evening. What was their status over the day, their overall load and their personal feelings.” **Head coach/Manager 1** “Always before the training, they have to tell me a number from 0 to 10 on how fit they are. Then again, right after the training and again two hours after the training. Then, I have a bit of an indication of how they are doing or whether they are reaching the limit. Indeed, with this information, I then adapt the planning and, if needed, adjust it.” **Former athlete, and current physiotherapist and S&C coach** “There are essential and ongoing conversations between the athlete and the coach. The athlete-coach relationship in free skiing and snowboarding, I believe, is particularly important because sometimes the coach must encourage the athlete to go for something that might be scary. Equally, the athlete must have a complete understanding that the coach will sometimes say, “*Not today, still not right*”. So, that’s the monitoring of what’s going on.” **Sports psychologist**

*S&C* strength and conditioning

**Table 3:** Concepts, subcodes and exemplary quotes on goal setting and planning through shared decision making

Main concept	Subcode	Exemplary quote
Planning before doing: goal setting and shared decision making	Testing data informs on the decision-making process	“It could be that we believe these tests indicate how these athletes respond to this type of training. […] So, it can help to get some baseline data, but also inform on the training and help to guide us through the process.” **S&C coach 3** “We get an idea of how our program has been geared (with testing), […] and we can adjust the program.” **Head coach/Manager 2** “They come to the results (of the testing assessment) – having an insight that they see what kind of work still needs to be done. Then you talk about it with each other. What the goals are, what still should be improved, what is good and should be further strengthened, where there is still a need, what is good, or what you should have in skiing.” **Former athlete, and current S&C coach** “We have a system of lights: red, orange, yellow and green. It’s all individualized. If anything lights up, we will address and adapt their training based on what we have found to ensure they don’t develop any injuries. If training is not moving the way it should, we’ll get this addressed individually.” **Physiotherapist 3**
	Individualised planning: teamwork effort putting the athlete in the centre	“The athlete is involved in goal setting and planning. I want the athlete to own the program. I want the athlete to be the one who’s doing what they want to do, and then, we’re talking back and forth. So, we’ve got an overall plan that says, “*This is where you are*”, and then we know what we will do this season and where we want to go.” **Sports psychologist** “Although we are a very large team, we try to be very athlete specific. […] The coach who is dealing with the athlete understands that that single athlete is the most important person. So, they might have a group of eight athletes, and each athlete should think they are the most important one.” **Head coach/Manager 2** “They are all different, so they will all have different programs based on their form and aims.” **Physiotherapist 1** “It depends on the athlete and their individual scenario and context.” **S&C coach 1** “Anything that comes out from testing that lights up needs more attention. It’s individualized. And then, there are the coaches who build the program based on what they believe is needed, together with physiotherapists and S&C coaches. Adapting each individual program based on what came from the screenings to make sure that everything can work together and is adapted to everyone. And we try, of course, to optimize this.” **Physiotherapist 3** “You have to listen carefully to the athletes. You have to get to know them and communicate with them. This is, to me, the most important thing in our sport.” **Head coach/Manager 1** “Yes, of course, I work with the on-snow coaches, and I have to adapt the whole planning to the on-snow plan and then to the competition season. So that they are ready when the season starts. […] I think you have to put the athlete at the centre a bit more, leave out the other interests and then it will be fine. I think it’s natural that everyone tries to make their own garden, but ultimately, the athlete has to be the centre of attention to get the best for him.” **Former athlete, and current physiotherapist and S&C coach**
	Structured but flexible: the planning needs to be adaptable	“Knowing the athlete. Suppose you have a good relationship with the athlete. In that case, you know when they are not quite on it, or you can use your experience to see that maybe the athlete is experiencing a bit more physical and mental fatigue from competition, or training, or school or, relationships, or whatever. So, you can make little subtle changes based on that. […] I think having a clear overview of the big picture of the snow sports and what they’re working towards, there is where you can help the athletes in that way.” **S&C coach 1** “Elite athletes have coaches available, and they can change training programs from day-to-day. Variation is an important part of motivation, but load management is also important.” **Physiotherapist 1** “The premise was always staying healthy and the dialogue with the athletes. The exchange that takes place: what is the goal? Where do we want to go? If we don’t achieve the goals, we discuss it with the athlete, more on what was wrong in my planning rather than what the athlete did wrong, and how we can achieve the goals together as a team or whether we have to change the plan or approach with constant dialogue between coaches and athlete.” **Head coach/Manager 1** “There is always an interview between the medical team, the athlete and the coaches to identify things to work on, and then for goal and aim setting as well.” **Physiotherapist 1**

*S&C* strength and conditioning

**Table 4:** Concepts, subcodes and exemplary quotes on closing the training plan and the ongoing process

Main concept	Subcode	Exemplary quote
Closing the cycle: implementing the training plan and starting it over	The training programme itself	“The goals are, of course, individual, depending on the age group or the level of the athletes. We have different goals in the summer season, so for me, it was always important to develop the athlete physically and mentally.” **Head coach/Manager 1** “You can kind of have small windows where you try to do and make the best for that scenario or that context. […] In my opinion, it’s better to have something that you can do consistently over a period of time as opposed to a program that looks really good on paper. Still, maybe you’re not able to do it properly because you don’t have access to that type of equipment.” **S&C coach 1** “So, I think most of our head coaches and coaches are very in tune to what we call thinking on the fly, thinking at the moment and being able to adjust. […] In summer, with a focus on athletic development, it is actually a premise for us to create multi-year plans as a development strategy for athletes. […] The specific goals are, of course, dependent on the athlete’s performance level, whether this is European Cup level or FIS level.” **Head coach/Manager 2** “There are always things happening. So, then the training program has to be adapted.” **Physiotherapist 1** “But, of course, you also have to make a few adjustments, and you have to do that all the time in winter with the planning, the races, the training sessions, and then again the European Cup/World Cup. That is stressful, but of course, you try to do the best you can. But it’s not always 100% possible all the time, that’s not possible. And that’s why they have to be flexible. […] You set it (training plan) up together: short-term, long-term, and medium-term. And you can pursue them if you stay healthy; otherwise, you can go back.” **Former athlete, and current S&C coach**
	Injury prevention is part of training	“We need values, values, and values, always trying to get better, whether it’s maximum strength, explosive strength, endurance, […] it’s actually just injury prevention for us. […] The most important thing is that the athlete skis without pain. And then there is a lot to do to make it possible at all. […] What is happening in other sports and the methods used are pretty good, so maybe we can apply them in snow sports, but it’s really hard to say. We definitely need more research into mechanisms behind certain injuries in snow sports and then put interventions into place to see if that affects those injuries. It’s a super interesting area, but there’s a lot unknown. Sort of a cloudy area, should we say?” **S&C coach 2** “Especially with skiers, I want them to be very, very strong in the legs. […] I want all their muscles surrounding the knee to be really strong. […] I think there are probably a few skiers out there that are so good technically that they can get away with being weak, but I think the vast majority of the skiers must be very strong in the lower body and in the core”. **S&C coach 3** “If nothing else, we are making a good, well-rounded athlete. We try out to really think about this (injury prevention) in April, May, June, that sort of time, post-season.” **Head coach/Manager 2** “We certainly have the ISPA injury prevention program that we include in our training program. Then, we try to be more specific, like doing things in the warm-up area. We try to include all these things. The ones that are also scientifically meaningful that bring you something.” **On-snow coach** “No, we know no specific program. We are kind of making it up as we go.” **Sports psychologist**
	Training the mind	“On day X, it often depends on the mental state that the person dares to be the fastest or to take a certain risk at certain points. And then simply be fast. I believe that the mental state is very important, and I have also experienced this with the athletes.” **Physiotherapist 2** “If you get the motivation to push yourself to the limit every time, then you’ll make more progress. That’s important.” **Former athlete, and current physiotherapist and S&C coach** “If I’m a skier and I go down the Lauberhörn or The Streif in Kitzbüel, and I’m thinking about my dinner tonight instead of the course, and I make a mistake, I can kill myself. That is an extreme situation, but the risk is very high.” **S&C coach 3** “I’m focused on performance, I’m focused on the execution, in the pressure of competition, and I’m focused on getting them to a situation where they don’t push too hard, too fast at the wrong time.” **Sports psychologist** “Mental recovery is extremely important when you are in the speed area because when you are mentally overwhelmed, it means a high risk. A mistake is usually the end of the story, or it becomes a huge problem. In the speed area, you must not make any mistakes, neither in the preparation nor in the race itself. That usually ends with an injury.” **Head coach/Manager 1**
	Challenges within the competitive snow sport context: evidence base and contextual factors	“I know from my experience as an athlete that you have to find your environment yourself. And as an older athlete, you will have it. You then have all these paths that you have to go. For example, it is difficult if I want to go to the physical therapist since it’s a lot decentralized. I think it would be easier for young people, in particular, to have a good path from the start without having to gather everything together until they finally have it. […] For the young athletes, having something like a guide is key.” **Former athlete, and current physiotherapist and S&C coach** “Training is still under-researched in our discipline, and we’re still trying to find the answers to what is important and essential to support the riders in doing what they do. And keep them injury-free and strong enough to do what they are doing. So, a lack of information and data is the biggest trouble.” **Head coach/Manager 3** “There aren’t really any guidelines. Free Solo.” **S&C coach 2** “You can kind of have small windows where you try to make the best for that scenario or that context. […] In my opinion, it’s better to have something that you can do consistently over a period of time as opposed to a program that looks really good on paper. Still, maybe you’re not able to do it properly because you don’t have access to that type of equipment.” **S&C coach 1**

*S&C* strength and conditioning

### Testing and Training Are Needed to Achieve the End Goal of Winning

Participants described “winning” as the end goal of the testing and training, which requires athletes to compete in their best condition. Two main and interlinked targets were reported, namely performance enhancement and health protection, while different strategies were defined.

#### Goals Are Driven by Physical and Mental Health

Performance goals include preparing athletes to increase their performance levels by improving their physical capacities, acquiring skills, especially in free skiing, and working on their mental abilities. The latter, with the leading support of their teams, entails dealing with pressure in their personal and professional domains and coping with risks, both in their training and racing settings. Subsequently, preparing and supporting the athletes ensures they will “*get from A to B, from start to finish, as quickly as possible*”. Likewise, all the stakeholders acknowledged that a fit and healthy athlete is ready to perform. While they recognised their performance-driven environment, they agreed that health is a premise for optimally performing. They perceived injury or pain as a hurdle to perform, stating that as long as the athlete is healthy, coaches and team staff can push them into their best version. Consequently, participants highlighted this as reason for injury prevention and health protection being training goals.

#### Mental Aspect is as Important as the Physical Aspect

When athletes and the staff were asked about the importance of mental health, training and abilities, they all acknowledged that the athletes’ mental aspects were just as important as their physical aspects. Coaches and team staff mentioned that athletes encounter different types of pressures and how they deal with them. For instance, they reported that athletes cope differently with performing under pressure, the nerves before a competition or the pressure to keep their spot within the team. These dimensions of pressure could come internally from themselves, their team, their family or from external sources, such as the National Association, media and sponsors. A mentally strong and self-confident athlete was considered to be a resilient athlete who dealt better with such dimensions of pressure and ultimately performed better. Similarly, athletes, coaches and sport psychologists agreed upon the importance of getting focused immediately before competitions and how elemental it is to be “*ready on day X*”.

#### Short-Term and Long-Term Goals of an Athlete’s Career Development

Coaches and team staff noted their key role in supporting and helping in athlete development. They recognised that such an approach encourages and assists athletes in developing themselves to become top athletes while being fit and healthy throughout the development process. The development path was described in short-term and long-term views, in which the work carried out during a whole season was deemed from a short-term perspective, whereas the long-term development course was aligned with an athlete’s career. Moreover, the team around the athlete also mentioned that training goals must be adapted to young athletes. The main focus is on introducing them to on-snow and physical training involving fun activities, with the long-lasting aim of preparing them for skiing while underlining the crucial role of preventing injuries.

### Why is Assessing the Athlete’s Status Fundamental?

Based on the stakeholders’ perspectives, assessing an athlete’s status periodically led to the collection of data on their physical and mental status by different means, eventually guiding the next steps in the training plan process.

#### What is the Status Quo of the Athlete? Baseline and Follow-Up Assessments

Participants employed both objective and subjective testing measures. Coaches and team staff mentioned that they perform objective assessments twice a year with elite athletes. Although different terminology was used, these periods coincided with the end of one season and the pre-season period of the upcoming season. The stakeholders emphasised that both assessments are used not only for testing performance but also for informing health threads and, in some cases, for both purposes. Objective testing measures generally included a battery of tests consisting of a medical check focusing on the cardiovascular, musculoskeletal and visual systems. They also tested for physical capacities (e.g. strength, mobility, power, endurance and balance). Likewise, the stakeholders mentioned conducting subjective assessments to identify an athlete’s physical and mental weaknesses and strengths through tests, self-reported questionnaires, apps and interviews.

#### There is a Critical Need for the Adequate Tests, Criteria and Normative Values

Coaches and team staff mentioned that the criteria by which these assessments are carried out fall under the consensual idea of “*what they have always done*”. Different factors, such as S&C coaches’ and medical staff’s own experience, scientific literature, background and education, discussions with other S&C coaches and physiotherapists from other teams, and trial and error cycles, play a role when defining these criteria. Participants outlined the limited literature on objective and subjective assessments, including uncertainties on what testing measures to perform and their reasoning, the lack of reference and normative values for physical capacity tests (e.g. strength and endurance), and the differences between sexes. Moreover, they highlighted a shortage of knowledge regarding how athletes have reached their full potential. Thus, coaches and team staff acknowledged the need for adequate tests to assess performance and health. Regarding young athletes, coaches and team staff highlighted that their assessment depends on the athlete’s club or region and a lack of a system in place. Coaches reported that their tests for youth were based on a score scale across different tests assessed by coaches.

#### Monitoring as the Approach of Repetitive Testing All Season Long

While some test assessments occur once or twice a year, other measurements, framed under the umbrella term of monitoring measures, occur throughout the season at different timepoints. Monitoring was identified as continuous testing throughout the season by all the participants involved in assessing the athlete’s status. Monitoring methods include tools such as a subjective movement analysis, questionnaires and other tracking techniques to measure load data. These loads encompassed tangible physical and on-snow training loads combined with the self-reported rate of perceived exertion (before, immediately after and after some hours of training), sleep quality, overall feelings and mood, and fatigue (physical and mental).

### Planning Before Doing: Goal Setting and Shared Decision Making

All participants, particularly athletes, coaches and team staff, mentioned that goal setting and planning involve two pillars: communication and shared decision making. They were united when foregrounding the significance of an athlete-centred process supported and driven by building strong relationships within the team and their influence on effective and open communication.

#### Test Data Informs on the Decision-Making Process

In this respect, they defined goal setting as the ongoing process that originates with the test results and ends with establishing the athlete’s goals. All the collected outputs on the athlete’s status are analysed and discussed by the coaches and team staff who work around the athlete, which eventually leads to informing further steps of the decision-making process. Communication and teamwork efforts were described as essential during the goal-setting stage. Coaches and team staff, in particular, underscored the power of baseline testing and monitoring data in goal planning, as they add valuable meaning in different dimensions, such as guiding future steps, athletes’ responses to training, guiding rehabilitation in cases of injury and baseline values in return-to-sport, and athletes’ fatigue.

#### Individualised Planning: Teamwork Effort Putting the Athlete in the Centre

Understanding the athlete and the snow sports context, explaining to athletes and helping them understand what, when, where, and most importantly, why they do so was pivotal for establishing a shared decision-making system. Altogether, they insisted on a continuous cyclic planning approach that embraces all stakeholders assisting in designing and agreeing on individual goals that meet the athlete’s needs and personal goals. Most of the time, the stakeholders involved in this steady and systematic course were athletes, head coaches or managers, on-snow coaches, S&C coaches and physiotherapists. Moreover, other professionals who may participate in the process include sports psychologists (or mental trainers), sports medical doctors, sports scientists and nutritionists.

#### Structured but Flexible: Planning Needs to be Adaptable

Depending on these individual goals, they described planning as being devised into different scenarios, which ranged from day-to-day to long-term macro plans. Participants also mentioned that gathering data provides a perspective on the individual context of the athlete, ultimately promoting continuous adjustments and adaptations to the athlete’s training plan, in which communications play a key role. The eventual outcome of the systematic sequence of testing and training is the development of a tailored and adjusted training plan for the athlete’s requirements, defined by and based on objective and subjective testing data, including, for instance, the weaknesses and strengths identified through subjective testing. Furthermore, they insisted on the flexibility of the training plan in terms of tailoring and fine-tuning it to the different stages within a season, travelling and changes throughout the season (e.g. calendar), weather and snow factors, access to facilities and equipment, and most importantly, to the constant athlete’s status and potential injuries. However, despite all these restraints, they stated that they “*try to do and make the best for that scenario or context*”.

### Closing the Cycle: Implementing the Training Plan and Starting it Over

Coaches and team staff involved in training described the process as a cyclic pathway, which can be established in a short-term period (e.g. a whole season, including both summer and winter) and in a long-term period (e.g. an athlete’s career). Eventually, training planification informs for and leads to the starting point of the process, which is characterised by finding a balance between enhancing performance while protecting it and the ultimate goal of winning.

#### Training Programme Itself

The underlying aims of training structures rely on increasing an athlete’s physical, skill and mental capacities while reducing health and injury threats that might be identified through testing or may occur during their training planning. Furthermore, coaches and team staff mentioned that the training structure could vary depending on the time within the season. For example, summer training is commonly characterised by large loads of physical training, also known as dryland training, combined with progressive on-snow training. This training block usually takes place overseas and in the Southern Hemisphere. In contrast, during the winter, on-snow training is more predominant than physical training. Coaching staff also highlighted differences between dryland and on-snow training. Whereas dryland training includes strength, endurance and power training, skill and movement patterns training (e.g. landings, tricks), and mental training, on-snow activities range from freeskiing, skill on-snow training, technical drills (e.g. in alpine skiing rotatory, edging and pressure drills), performing tricks, skiing patterns, and runs and equipment testing in different situations (e.g. terrain steepness, course design difficulty) and under varying snow conditions (e.g. icy, hard, soft). Connected with the challenges they faced during the whole season, all the stakeholders acknowledged the limitations they encountered, pointing out that those regarding physical training were less burdensome than those related to on-snow training.

#### Injury Prevention is Part of Training

According to all the stakeholders’ perspectives, testing and monitoring approaches, goal setting and training are also considered parts of injury prevention interventions to protect an athlete’s performance. Hence, the training plan also includes injury prevention strategies, including physical and skill training, with a special focus on the build-up period during the off-season, the aforementioned monitoring approaches and load management tools, and exercise-based interventions addressing the weaknesses identified in subjective testing. For example, physiotherapists and S&C coaches referred to an alpine skiing-specific injury prevention programme focusing on young athletes (e.g. “Injury Screening and Prevention—Alpine Skiing” (ISPA) intervention). They suggested that they make use of 11 + warm-up programmes from the football setting modified to their snow sports contexts. However, they also reiterated the need for additional research on injury prevention, especially concerning injury prevention guidelines or programmes, as they “*make it up as they go*”, trying their best with their knowledge and experience.

#### Training the Mind

Regarding mental training, participants mentioned that the ability to focus can be learned through daily practice with the primary help of a specialist (e.g. sports psychologist) or, otherwise, with other non-specialist team members. Furthermore, all stakeholders dwelled on risk-taking and risk-management behaviours. They acknowledged that the different disciplines of these competitive snow sports deal with different inherent and sport-specific risks. For instance, risk-taking behaviours rely more on trick performance in snowboarding and freestyle skiing subdisciplines, while in alpine skiing and some subdisciplines of snowboarding and freestyle skiing risk-taking behaviours relate to speed. However, they emphasised that athletes need to take risks to be fast or to perform the best tricks, while viewing it as a learning process in which communication and trusting relationships between athletes and coaches are essential.

#### Challenges Within the Competitive Snow Sport Context: Evidence Base and Contextual Factors

Participants reported a lack of evidence, guidelines and protocols related to training methods in competitive snow sports. Strength and conditioning coaches, coaches and other team staff referred to approaching such limitations as a “*trial and error*” cycle, in which they eventually overcome these challenges by using their own criteria and experience. Additionally, they underlined challenges they encounter in their daily practice, both in physical and on-snow training. These entailed shared factors for both environments, such as access to facilities, lack of qualified personnel, funding, equipment and injuries. In contrast, while decentralisation of infrastructures and long distances were described as issues in terms of physical training, on-snow training had limitations such as weather and snow factors, as well as travelling and training overseas.

## Discussion

This qualitative study outlined testing and training practices and perspectives from athletes, coaches, head coaches and managers, and healthcare providers in the competitive snow sports context. Our findings describe how performance enhancement and health protection are intertwined within the systematic, cyclic, individualised, and flexible concepts of testing and training. These include an athlete assessment, goal setting and shared decision-making processes, and training plan implementation as key approaches to ensuring an athlete’s optimal preparation to perform in such sports settings.

### Performance and Health as Pillars to Shape Athletes’ Winning Paths

The current study provided insight into the integrated relationship between athletes’ performance and safeguarding their health, as a single concept. While snow sports are well known for their performance-driven environment [1, 19], whose ultimate goal is winning, protecting an athlete’s health has also become paramount [4, 16, 27, 39]. Such a balanced approach not only enhances performance but also helps to preserve health and minimise the risk of injury. In this direction, participants described an integrated approach motivated by performance-centred goals yet using health-driven strategies [17, 39], focusing on physical and mental training, protection and prevention [40]. In addition, the previous literature has shown that injuries have a detrimental effect on performance and athletic success [41]. To best serve athletes, an integrated performance system can assist in ensuring performance success and the athlete’s development both in the short term and long term, as remarked upon by participants. For instance, in the run-up to the 2012 London Olympic and Paralympic Games, the UK Athletics developed “The Integrated Performance Health Management and Coaching Model”, where health and performance departments operated in synergy [17]. Considering that athletes and their staff highlighted that the main goal is to be optimally prepared to perform and win, the medical team staff and the coaching and performance team staff must work closely. Likewise, the medical team and coaching staff acknowledged no black-and-white separation but a greyscale continuum regarding injury risk mitigation strategies and performance development. Both constructs operate conjointly, targeting physical and mental aspects, as they are part of the training structure itself [4, 40]. In this regard, Gabbett proposed the “training-injury prevention paradox”, suggesting that hard and smart training may act as a “vaccine” to protect against injuries [13]. Therefore, intertwining and managing athletes’ performance and health is imperative to stepping onto the top of the podium. Future research should explore this relationship in greater depth and how it can be optimised in testing and training within the snow sports context.

### “It All Begins at the Starting Gate”: Athletes’ Assessment and Monitoring

Our findings showed that data on athlete status are gathered through assessing and monitoring them. Knowing who the athlete is from a performance and health perspective was considered central because it eventually informs athletes of individualised goal setting and goal planning. Hereafter, the process moves toward developing a training programme, covering all the dimensions of the athlete, from the physical to mental aspects, from basic to sport-specific aspects and from performance to health. Accordingly, it has been advocated that setting goals in sports settings leads to enhanced performance [42]. Indeed, monitoring was defined as a constant and regular form of testing both performance and health, differentiating between external and internal loads or between physical, psychological and social loads [6, 9, 11, 43]. Recently, including in snow sports settings, athlete load monitoring has become a regular practice for determining the “dose–response” relationship to competition and training loads, considering objective measures, subjective outcomes, psychological measures and lifestyle-related factors [4, 6, 43]. All these monitoring tools were employed to inform practitioners about practice, and they were defined as extremely valuable as they guided future actions. In this respect, West et al. outlined five overarching levels in which training loads communicate about an athlete’s status, which ranged from long-term to short-term decisions (e.g. long-term use, season planning, day-to-day planning. in-season adjustment, and feedback) [6]. In contrast, Dijkstra et al. presented a continuous health monitoring system in which health and performance risks are graded as a result of an athlete’s health status [17]. Hence, monitoring athlete loads can assist in increasing stakeholders’ knowledge of their responses and adaptations to improve and adjust athletes’ training plans while enhancing performance and reducing injury risks [3, 12]. While some data are available for specific disciplines and contexts (e.g. Austrian and Swiss alpine ski racers and snowboarding) [9, 21, 44], as arose by participants, there is a lack of specific understanding of the physical and mental demands and training requirements of sport-specific demands. Consequently, considering the relevance of this topic and the uniqueness of the context, there is a need for appropriate tests, criteria, and normative values for testing and monitoring in the snow sports context.

### Goal Planning and Setting: A Cornerstone Leading to the Training Plan

Before implementing the training plan, the last step hinges upon establishing a shared decision-making process driven by communication among all team members to meet the athlete’s needs. As a result, different aspects come into play when designing and developing a training programme, particularly understanding the athlete’s individual needs, contextual factors, communication and shared decision making [1, 19, 39]. Therefore, planning should be adjusted to meet the needs of athletes to cope with their physical, psychological, and social loads and to adapt to travel loads and tight schedules [6, 11]. For instance, a biopsychosocial and interdisciplinary approach may put athletes at the centre of the process and imply interprofessional communication and collaborative actions across different team members [45]. Furthermore, the context and changing environments and conditions characterise and influence competitive snow sports. As such, various contextual factors may play a role in training. Athletes’ plans should be contextualised in their environment to inform their process. In this regard, data collection through assessing and monitoring athletes continuously contributes to building an athlete’s holistic picture. Embedding both physical and mental athlete periodisation into the daily structure of the action plan nourishes and provides dynamism and flexibility to the training plan, which was highlighted when tailoring it to an athlete’s needs [46]. Factors related to athletes (e.g. physical and athlete characteristics), coping strategies, the environment, and racing and training facilitate the shaping and further implementation of such a comprehensive plan [6, 40]. In addition, the strength of communication and shared decision making relies on fitting and fine-tuning the training plan and on the team around the athlete and athletes themselves, who all together make informed decisions about its structure and periodisation [19, 39, 46]. Hence, through effective communication, teamwork and an integrated decision-making process, every team member has a role and contributes to the unified goal of preparing the athlete in their best condition to perform and win.

### Implementing the Training Plan: The Last Piece of the Puzzle

The final stage of the cyclic process described by participants is developing and implementing the training plan. This wheel pattern has also been shown with the “training-process framework”, where adaptations in the training plan constantly take place [19]. As mentioned above, collaboration and open communication among team members promote integrated performance support, which is of the utmost relevance in establishing technical and tactical strategies while safeguarding athletes’ physical and mental health [17, 39]. Furthermore, the recent literature highlighted the need to introduce risk management strategies as part of the injury prevention behaviours included in training programmes [47]. This risk management approach is described as the final stage of a decision-making process. In this regard, the integration of physical and mental interventions by improving athletes’ knowledge, awareness and skills (e.g. body and mind), health, injury prevention, risk taking, and the balance between pushing limits and safety leads to the shaping and implementation of risk management behaviours in their current practice [47]. In connection with the particularities of each of these competitive snow sports, their on-snow and off-snow (or dryland) training activities may differ between them. In freestyle skiing and freestyle snowboarding, athletes seem to place less attention on strength training and more onto physical and mental skill and technical training, thus more orientated to developing their skills and tricks [46]. In contrast, in alpine skiing and the other speed disciplines among freestyle skiing and snowboarding, athletes and their supporting team prioritise strength, endurance and skill training. Taken together, athletes across all disciplines deal with a particular risk-taking component [46, 47]. Hence, these findings can contribute to setting the stage for future work in terms of on-snow and dryland training, both from the perspectives of performance optimisation and prevention efforts. Thus far, although physical and mental training periodisation has been documented in competitive alpine skiing, snowboarding and freestyle skiing [1, 7, 46], little is known about competitive snow sports, accentuated by the different demands across disciplines. Therefore, acknowledging the importance of flexibility and how contextual factors can affect the training structure, further research should focus on elaborating on-snow and off-snow training plans and structures and understanding each discipline’s physical and mental demands to develop and implement more sport-specific interventions.

### Strengths and Limitations

The use of diverse methods strengthened the trustworthiness of our findings [48]. Data and investigator triangulation were applied for credibility by incorporating different stakeholders, such as athletes, on-snow and off-snow coaches, managers and healthcare professionals. The data analysis process included researchers from various backgrounds and cultures. We recognise that one main researcher providing contact (JS) might have coloured the sample and influenced the data collected and the study results. Nevertheless, applying qualitative methods facilitated uncovering nuanced insights into testing and training practices in such a context. Initially, three independent researchers (OBM, CB and PB) conducted the initial part of the data analysis. After that, two researchers (OBM and CB) conducted the remainder of the data analysis. Multiple meetings and discussions took place to evaluate the analysis and its relationship to the previous literature to increase confirmability and demonstrate the quality of the research. Regarding transferability, study participants included elite alpine skiing, snowboarders and freestyle skiing athletes/staff competing in FIS events from international teams from the European, Asian, North American and Oceanic continents, including countries from both hemispheres. The study sample included mostly male individuals, while female individuals were underrepresented. According to resources and sports culture, this scenario coincides with the reality of competitive snow sports staff. However, such a diverse sample with different experiences, backgrounds, countries and cultures about testing and training thoroughly describes the topic. Furthermore, this study only included three disciplines among all competitive snow sports disciplines. Therefore, we acknowledge that our findings are context specific and circumstantial and that comparisons with other sports contexts should be cautiously made. Dependability was accomplished by creating an audit trail in which memos were written to record the development and reporting of the findings.

## Conclusions

The ultimate goal of testing and training practices in competitive snow sports is winning. Our study provided insight into the role of testing and training within the competitive snow sports setting. Performance and health act conjointly toward optimally preparing an athlete to achieve their ultimate goal of winning. The derived concepts provide a comprehensive view of the development process of the systematic, cyclic, individualised and flexible training plan, starting with assessing an athlete’s status and finishing with the eventual training plan, where monitoring tools, communication and collaborative decision making play major roles.

## Supplementary Information

Below is the link to the electronic supplementary material.

Supplementary file1 (PDF 82 KB)

Supplementary file2 (PDF 207 KB)

## Declarations

### Availability of Data and Material

Data are available upon reasonable request; however, key data are anonymously shared in the results, and no data with the potential to identify participants, such as audio files, can be shared.
