# Supplementary material for: “Health is Just the Basic Requirement for Optimal Performance and Winning”: Stakeholders’ Perceptions on Testing and Training in Competitive Alpine Skiing, Snowboarding and Freestyle Skiing
Source: Sports Med. 2024 Sep 13;55(1):221–39. doi: 10.1007/s40279-024-02106-0 (PMC11787165; doi:10.1007/s40279-024-02106-0)
Supplement: Supplementary file 1 — Supplementary file1 (PDF 82 KB) [file 40279_2024_2106_MOESM1_ESM.pdf]

Supplementary material 1

"Health Is Just the Basic Requirement for Optimal Performance and Winning" – Stakeholders' Perceptions on Testing and Training in Competitive Alpine Skiing, Snowboarding and Freestyle Skiing

Journal: Sports Medicine

Authors
Oriol Bonell Monsonís[a,b], Peter Balsiger[c,d], Evert Verhagen[a,b], Vincent Gouttebarge[b,e,f], Jörg Spörri[c,d], Caroline Bollinga[e]
a. Amsterdam Collaboration on Health & Safety in Sports, Department of Public and Occupational Health, Amsterdam Movement Sciences, Amsterdam UMC, University Medical Centres – Vrije Universiteit Amsterdam, Amsterdam, the Netherlands.
b. Amsterdam Movement Sciences, Musculoskeletal Health & Sports, Amsterdam, The Netherlands.
c. Sports Medical Research Group, Department of Orthopaedics, Balgrist University Hospital, University of Zurich, Zurich, Switzerland.
d. University Centre for Prevention and Sports Medicine, Department of Orthopaedics, Balgrist University Hospital, University of Zurich, Zurich, Switzerland.
e. Amsterdam UMC location University of Amsterdam, Department of Orthopedic Surgery and Sports Medicine, Meibergdreef 9, Amsterdam, The Netherlands.
f. Section Sports Medicine, Faculty of Health Sciences, University of Pretoria, Pretoria, South Africa.

Corresponding author
Oriol Bonell Monsonís. E-mail address: o.bonellmonsonis@amsterdamumc.nl

Supplementary material 1

Consolidated criteria for reporting qualitative studies (COREQ): 32-item checklist.

Developed from: Tong A, Sainsbury P, Craig J. Consolidated criteria for reporting qualitative research (COREQ): a 32-item checklist for interviews and focus groups. *International Journal for Quality in Health Care*. 2007. Volume 19, Number 6: pp. 349 – 357

No Item	Guide questions/description		Reported on
**Domain 1: Research team and reflexivity**			
Personal Characteristics			
1. Interviewer/facilitator	Which author/s conducted the interview or focus group?	Peter Balsiger	Data Collection
2. Credentials	What were the researcher's credentials? e.g., *PhD, MD*	MSc	Reflexivity
3. Occupation	What was their occupation at the time of the study?	Master student	Reflexivity
4. Gender	Was the researcher male or female?	Male	Reflexivity
5. Experience and training	What experience or training did the researcher have?	PB is a Swiss human movement scientist and an athletic coach.	Reflexivity
Relationship with participants			
6. Relationship established	Was a relationship established prior to study commencement?	No	Not reported

7. Participant knowledge of the interviewer	What did the participants know about the researcher? e.g., *personal goals, reasons for doing the research*	General outlines of research as described in information letter sent by email.	Participants

8. Interviewer characteristics	What characteristics were reported about the interviewer/facilitator? e.g., *Bias, assumptions, reasons and interests in the research topic*	Interviewer was an athletic trainer.	Reflexivity
**Domain 2: study design**			
Theoretical framework			
9. Methodological orientation and Theory	What methodological orientation was stated to underpin the study? e.g., *grounded theory, discourse analysis, ethnography, phenomenology, content analysis*	Grounded theory/ Concepts of Grounded Theory were applied such as the theoretical sampling (maximum variance during data collection), the constant comparison analysis and the exploratory nature of the study from a constructive interpretivist paradigm.	Study design
Participant selection			
10. Sampling	How were participants selected? e.g., *purposive, convenience, consecutive, snowball*	Main contact (JS) who worked closely with FIS recruited potential participants, being different stakeholders such as athletes, head coaches and managers, on-snow coaches, strength and conditioning (S&C) coaches, physiotherapists, and sport psychologists.	Participants
11. Method of approach	How were participants approached? e.g., *face-to-face, telephone, mail, email*	By email.	Participants
12. Sample size	How many participants were in the study?	13	Results

13. Non-participation	How many people refused to participate or dropped out? Reasons?	None.	Not reported
Setting			
14. Setting of data collection	Where was the data collected? e.g., *home, clinic, workplace*	All interviews happened on a secure online platform according to the participants' availability.	Data collection
15. Presence of non-participants	Was anyone else present besides the participants and researchers?	No	Not reported
16. Description of sample	What are the important characteristics of the sample? e.g., *demographic data, date*	Limited demographics presented: gender, stakeholder role, high-performance snow sports discipline, country or representing country.	Results
Data collection			
17. Interview guide	Were questions, prompts, guides provided by the authors? Was it pilot tested?	Topic list and interview questions were pilot tested.	Data collection.
18. Repeat interviews	Were repeat interviews carried out? If yes, how many?	No	Not reported
19. Audio/visual recording	Did the research use audio or visual recording to collect the data?	Audio-recorded	Data collection
20. Field notes	Were field notes made during and/or after the interview or focus group?	Yes, during and after.	Not reported

21. Duration	What was the duration of the interviews or focus group?	The interviews had an average duration of 46 minutes (ranging from 34 to 71 minutes).	Data collection
22. Data saturation	Was data saturation discussed?	Yes	Data collection
23. Transcripts returned	Were transcripts returned to participants for comment and/or correction?	No	Not reported
**Domain 3: analysis and findings**			
Data analysis			
24. Number of data coders	How many data coders coded the data?	3 coders.	Data analysis and Figure 1
25. Description of the coding tree	Did authors provide a description of the coding tree?	Yes	Data analysis
26. Derivation of themes	Were themes identified in advance or derived from the data?	Derived from the data	Data analysis
27. Software	What software, if applicable, was used to manage the data?	ATLAS.ti software (Scientific Software Development GmbH, Berlin, Germany; version 8.4.5)	Data analysis
28. Participant checking	Did participants provide feedback on the findings?	No	Not reported
Reporting			

29. Quotations presented	Were participant quotations presented to illustrate the themes/findings? Was each quotation identified? e.g., *participant number*	Quotes were presented to illustrate themes and findings. Each quotation was identified by participant function and number.	Results tables 1-4
30. Data and findings consistent	Was there consistency between the data presented and the findings?	Yes	Results
31. Clarity of major themes	Were major themes clearly presented in the findings?	Yes, four major themes are clearly presented.	Results, tables 1-4 and Figure 2
32. Clarity of minor themes	Is there a description of diverse cases or discussion of minor themes?	Yes, the minor themes related to the core categories (in less detail) are also presented.	Results, tables 1-4 and Figure 2
