# Supplementary material for: “Health is Just the Basic Requirement for Optimal Performance and Winning”: Stakeholders’ Perceptions on Testing and Training in Competitive Alpine Skiing, Snowboarding and Freestyle Skiing
Source: Sports Med. 2024 Sep 13;55(1):221–39. doi: 10.1007/s40279-024-02106-0 (PMC11787165; doi:10.1007/s40279-024-02106-0)
Supplement: Supplementary file 2 — Supplementary file2 (PDF 207 KB) [file 40279_2024_2106_MOESM2_ESM.pdf]

Supplementary material 2

"Health Is Just the Basic Requirement for Optimal Performance and Winning" – Stakeholders' Perceptions on Testing and Training in Competitive Alpine Skiing, Snowboarding and Freestyle Skiing

Journal: Sports Medicine

Authors

Oriol Bonell Monsonís[a,b], Peter Balsiger[c,d], Evert Verhagen[a,b], Vincent Gouttebarge[b,e,f], Jörg Spörri[c,d], Caroline Bolling[a,e]

a. Amsterdam Collaboration on Health & Safety in Sports, Department of Public and Occupational Health, Amsterdam Movement Sciences, Amsterdam UMC, University Medical Centres – Vrije Universiteit Amsterdam, Amsterdam, the Netherlands.
b. Amsterdam Movement Sciences, Musculoskeletal Health & Sports, Amsterdam, The Netherlands.
c. Sports Medical Research Group, Department of Orthopaedics, Balgrist University Hospital, University of Zurich, Zurich, Switzerland.
d. University Centre for Prevention and Sports Medicine, Department of Orthopaedics, Balgrist University Hospital, University of Zurich, Zurich, Switzerland.
e. Amsterdam UMC location University of Amsterdam, Department of Orthopedic Surgery and Sports Medicine, Meibergdreef 9, Amsterdam, The Netherlands.
f. Section Sports Medicine, Faculty of Health Sciences, University of Pretoria, Pretoria, South Africa.

Corresponding author

Oriol Bonell Monsonís. E-mail address: o.bonellmonsonis@amsterdamumc.nl

Supplementary material 2: Topic list and interview questions

Topic	Questions
Training	1. What are your goals in training? (What do you pursue with training?)<br>2. Why did you choose these goals?<br>3. How can you achieve these goals?
Planning and execution of training	4. How are trainings planned?<br>5. How are the training plans adapted? (Based on what criteria?)<br>6. What is the motivation behind the planning? Are there any guidelines to follow?<br>7. Who has an impact and responsibility on the planning?<br>8. Are there any deviations from the planning of the training and the execution?
Testing measures	9. What objective measures do you use to assess the athletes?<br>10. What subjective measures or screening do you perform to assess the athletes?<br>11. Why do you perform these assessments and tests?<br>12. What do you do with the results of the testing measures? Could you guide me through the process?<br>13. Who is responsible at every stage?
Future perspectives of training and testing	14. Which injury prevention strategies do you apply? Why do you apply these strategies?

	15. What do you consider when choosing the injury prevention strategies that you currently use?<br>16. How do you choose each strategy to apply, and why do you choose them?<br>17. What motivates you to apply an injury prevention strategy?
Injury prevention	18. What do you consider important?<br>19. What do you specifically do?<br>20. Are you aware of any injury prevention programs?
Mental aspects	21. What is the role of mental aspects?<br>22. Do you do anything to target these aspects?
